# Improvement of a Surfactant Blend for Enhanced Oil Recovery in Carbonate Reservoirs by Means of an Ionic Liquid

**DOI:** 10.3390/ijms24010726

**Published:** 2022-12-31

**Authors:** Nestor Tafur, Alberto P. Muñuzuri, Ana Soto

**Affiliations:** 1 CRETUS, Department of Chemical Engineering, Universidade de Santiago de Compostela, 15782 Santiago de Compostela, Spain; 2 Group of Nonlinear Physics, Galician Center for Mathematical Research and Technology (CITMAga), 15782 Santiago de Compostela, Spain

**Keywords:** blend, microemulsion, Winsor III, ionic liquid, surfactant flooding

## Abstract

The promising experimental performance of surfactant blends encourages their use in recovering the large quantity of crude oil still remaining in carbonate reservoirs. Phase behavior studies were carried out in this work to propose a blend for practical application. To that aim, the surfactants dioctyl sulfosuccinate sodium (AOT) and polyoxyethylene(8) octyl ether carboxylic acid (Akypo LF2) were mixed. A formulation consisting of 1 wt% of AOT_50wt%_/LF2_50wt%_ blend in synthetic sea water (SSW) led to a low value of interfacial tension with crude oil of 1.50·10^−2^ mN/m, and 0.42 mg/g_rock_ of dynamic adsorption. A moderate additional oil recovery (7.3% of the original oil in place) was achieved in a core flooding test. To improve this performance, the surface-active ionic liquid 1-dodecyl-3-methylimidazolium bromide ([C_12_mim]Br) was added to the system. The electrostatic interactions between the oppositely charged surfactants (AOT and [C_12_mim]Br) led to a higher surface activity. Thus, a formulation consisting of 0.8 wt% of AOT_20.7wt%_/[C_12_mim]Br_25.3wt%_/LF2_54wt%_ in SSW reduced the interfacial tension and surfactant adsorption achieved with the binary blend to 1.14 × 10^−2^ mN/m and 0.21 mg/g_rock_, respectively. The additional oil recovery achieved with the blend containing the ionic liquid was 11.5% of the original oil in place, significantly improving the efficiency of the binary blend.

## 1. Introduction

Although surfactant flooding has been studied and used as an effective enhanced oil recovery (EOR) method for sandstone rock reservoirs [1], the use of surfactants in carbonate rocks has been limited because of their high adsorption on the rock surface (anionic surfactants) and their inability to reduce the interfacial tension (IFT) to ultralow values (cationic and nonionic surfactants) [2,3,4,5,6]. Since carbonate rock reservoirs represent around 50–60% of the total oil around the world [7,8], several methods such as the use of low salinity water and nanoparticles are being tested for their exploitation [9]. However, the successful application in this kind of reservoir of the traditionally most efficient surfactants for EOR would be of high interest, which is why this has been the target of many studies. Among the tested possibilities, several works have taken advantage of the better performance of surfactant blends in comparison with their pure components used individually. These mixtures commonly show a synergistic effect on the reduction of interfacial tension between water and oil, and in some cases, surfactant adsorption on the rock surface is also reduced [10,11,12,13,14]. 

In our previous work [15], the phase behavior of three binary blends was studied at different salinity and temperature conditions. An anionic/nonionic blend was formulated with dioctyl sulfosuccinate sodium salt (AOT) and polyoxyethylene(8) octyl ether carboxylic acid (Akypo LF2). AOT is an anionic surfactant with a high lipophilic character [16,17,18]. Akypo LF2 is a triblock alkyl ethoxy carboxylate surfactant, tolerant to high salinity and long-term stable at high temperature, with a hydrophilic character [19,20,21]. Thus, a hydrophilic-lipophilic balance associated with a Winsor III behavior [22] was achieved blending both chemicals. In the same work [15], two anionic/cationic blends were formulated with AOT and surface-active ionic liquids (SAILs). These salts have been found promising for EOR applications [23,24,25,26,27]. However, they are usually not able to achieve ultra-low IFT by themselves. As far as we know, only one work can be found in the literature [28] where a Winsor type III system was achieved with a single SAIL: (S)-2-prolinolium dodecylbenzene sulfonate. Therefore, their blending with other surfactants is a technique that is gaining attention for EOR purposes [26,29,30]. Blends of AOT and 1-decyl-3-methylimidazolium chloride ([C_10_mim]Cl) or 1-dodecyl-3-methylimidazolium bromide ([C_12_mim]Br) led to Winsor III systems [15]. The AOT/SAIL blends showed high optimal solubilization parameters (good capacity to solubilize oil and water at the same proportion) but were unstable in the absence of oil, so they only could be injected in the reservoir as microemulsions. In contrast, the AOT/LF2 blend showed good stability in brine and lower sensitivity to blend ratio changes, but lower optimal solubilization parameters than the AOT/SAIL blends. 

The purpose of the present work is to evaluate the AOT/LF2 blend for EOR by means of core flooding tests, and also to test the possibility of improving the blend’s efficiency by adding SAIL [C_12_mim]Br. On one hand, this ternary mixture would take advantage of the strong electrostatic interactions between oppositely charged surfactant head groups of AOT and [C_12_mim]Br, leading to catanionic micelle formation with higher surface activity and greater capacity of solubilization than the individual surfactants [15,26,27,30,31,32,33]. On the other hand, the incorporation of LF2 would provide stability under harsh salinity and temperature conditions [19,20,21]. 

## 2. Results and Discussion

### 2.1. Experimental Section

#### 2.1.1. Blend Scans

**Figure 1:**
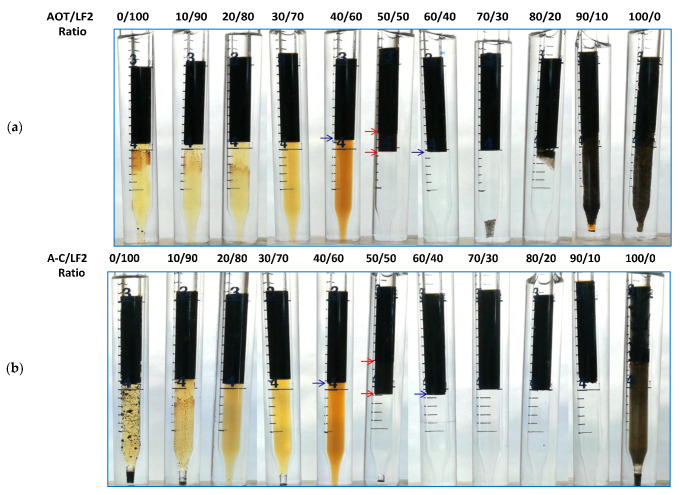
Blend scans at 25 °C: (**a**) AOT/LF2 and (**b**) A-C/LF2.

The surfactant LF2 and the ionic liquid [C_12_mim]Br are highly hydrophilic, showing microemulsion Winsor type I (according to Winsor’s classification) from 1 to 15 wt% NaCl and from 25 to 75 °C [15]. The surfactant AOT is highly lipophilic, being Winsor type II from 0.21 wt% NaCl [15,26]. The pseudo-component A-C (45 wt% AOT, 55 wt% [C_12_mim]Br) was found to be lipophilic, with a Winsor type II behavior in SSW at 25 °C (See Supporting Information, SI). Figure 1 shows the AOT/LF2 and A-C/LF2 blend scans at 25°C. Winsor type I microemulsions were obtained from 0 to 40 wt% AOT in the AOT/LF2 blend (Figure 1a). Winsor type III was observed at 50 wt% AOT, and the microemulsion shifted to Winsor type II at 60 wt% and higher AOT concentrations. Precipitation and high viscosity emulsions were observed for blends with high AOT proportions. A similar phase behavior was observed for the A-C/LF2 blend (Figure 1b), the difference being that the volume of the middle phase (shown in the figure between red arrows) was higher for this ternary blend. The behavior of the AOT/LF2 slightly differs from our previous study [15] where *n*-octane was used as the oil phase, and brine solution (5 wt% NaCl without divalent ions) as the aqueous phase. The Winsor III region found in tests with crude oil was slightly smaller. As expected, the capacity of solubilization of the blend is affected by the different compounds of the crude [12,17,32,34,35,36,37].

**Figure 2:**
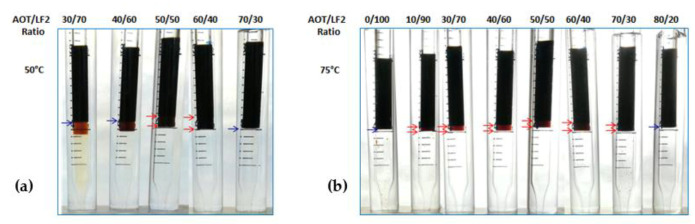
AOT/LF2 blend scans at (**a**) 50 °C and (**b**) 75 °C.

**Figure 3:**
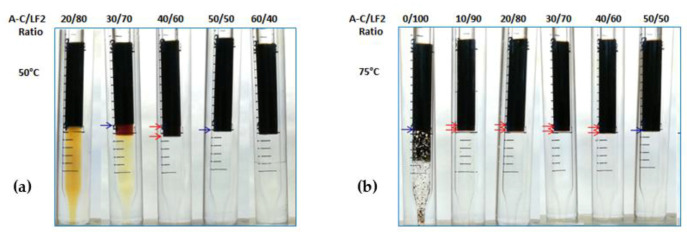
A-C/LF2 blend scans at (**a**) 50 °C and (**b**) 75 °C.

The blends were also evaluated at 50 °C and 75 °C. The results are shown in Figure 2 and Figure 3 for AOT/LF2 and A-C/LF2, respectively. In the case of the AOT/LF2 blend, the range of ratios where Winsor III systems were found increased with temperature. At 50 °C, triphasic systems were found from 50 to 60 wt% AOT, and at 75 °C, from 10 to 70 wt% AOT (Figure 2). However, the volume of the middle phase slightly reduced with temperature. Similar behavior was found for the A-C/LF2 blend: in a range between 10 and 40 wt% A-C, small Winsor type III systems were found (Figure 3). No further studies were carried out at 75 °C since both blends showed loss of efficiency to solubilize water and oil phases.

**Figure 4:**
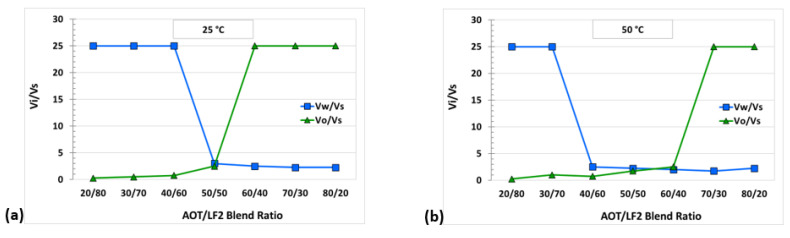
Solubilization parameters (*V_i_/V_s_*) for AOT/LF2 blend at (**a**) 25 °C and (**b**) 50 °C.

**Figure 5:**
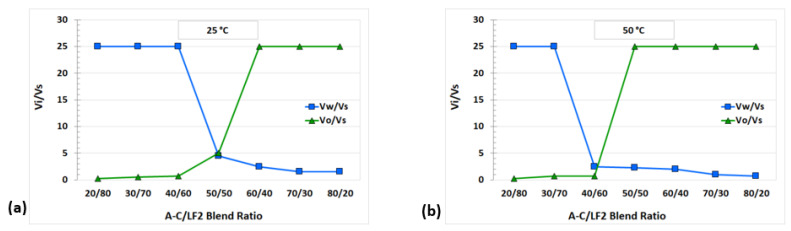
Solubilization parameters (*V_i_/V_s_*) for A-C/LF2 blend at (**a**) 25 °C and (**b**) 50 °C.

Oil (*V_o_/V_s_*) and water (*V_w_/V_s_*) solubilization parameters, as a function of the blend ratio, were calculated for both blends at 25 °C and 50 °C. A decrease in the optimal solubilization parameters (V_i_/V_s_*, *V_o_/V_s_* = *V_w_/V_s_*) with temperature was observed. The optimal solubilization parameters for the blend AOT/LF2 were 3 and 2.1 at 25 °C and 50 °C, respectively (Figure 4). In the case of the A-C/LF2 blend, V_i_/V_s_* decreased from 4.7 to 2.5 at the same evaluated temperatures (Figure 5). Table 1 shows the values of the optimal blend ratios, corresponding solubilization parameters, and estimated IFT by Huh’s equation, for both blends.

Table 1 shows a clearly different behavior of the blends studied regarding the influence of temperature on the optimal blend ratio. In the case of AOT/LF2, the estimated optimal blend ratio shifted from 50.2 to 55 wt% AOT when the temperature increased from 25 °C to 50 °C. The range of ratios where the blend behaves as hydrophilic increased with temperature due to a stronger interaction of the ionic head groups of the surfactants with the water molecules, as in the case of anionic surfactants. On the other hand, for the same temperature increase, the estimated optimal blend ratio for A-C/LF2 moved from 49.9 to 40.7 wt% A-C. Lipophilicity increased with temperature, as in the case of traditional nonionic surfactants. This last behavior was also observed for the blends AOT/[C_12_mim]Br and AOT/[C_10_mim]Cl in our previous study [15], and it may be explained by the reduction or cancellation of free electrostatic charges due to the high synergy between the oppositely charged head groups of the anionic surfactant and the cationic SAIL [31,32]. The surfactant LF2 is a weak carboxylic acid which behaves as a nonionic surfactant in neutral to acid solution [19].

#### 2.1.2. Stability Tests

**Figure 6:**
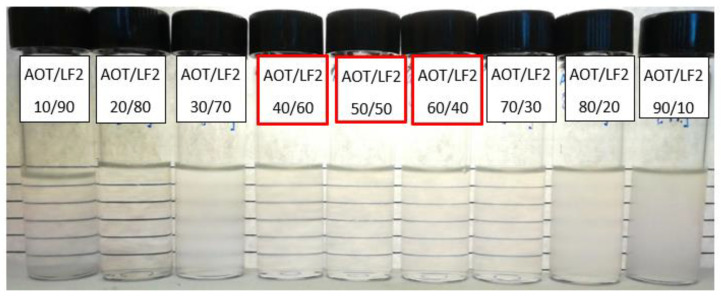
Stability test of aqueous formulations with 1 wt% AOT/LF2 blend (different ratios) at room conditions. The range of interest for further dynamic IFT evaluation is shown with red squares.

The stability of the aqueous formulations was initially evaluated at 1 wt% blend (AOT/LF2 and A-C/LF2) concentration at room temperature. During the evaluation, the A-C/LF2 concentration was reduced from 1 to 0.9 wt% to improve the stability of the blend. Figure 6 shows the results of this test with different AOT/LF2 ratios, from 10 wt% to 90 wt% AOT. Around the 50/50 blend ratio, the formulations remained translucent during the 4 weeks of the test.

**Figure 7:**
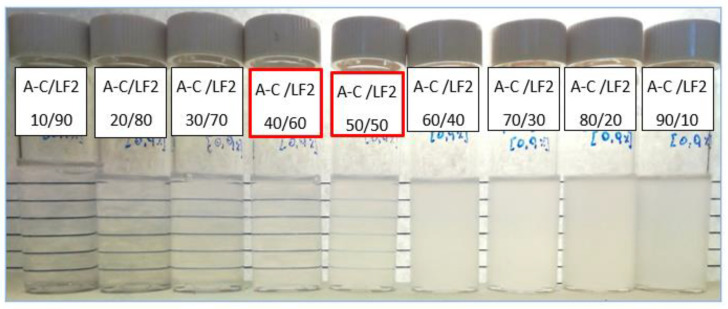
Stability test of aqueous formulations with 0.9 wt% A-C/LF2 blend (different ratios) at room conditions. The range of interest for further dynamic IFT evaluation is shown with red squares.

In the case of A-C/LF2, the stability evaluation was performed from 10 wt% to 90 wt% A-C. The blend remained translucent during 4 weeks in the range of interest from the 40/60 to 50/50 A-C/LF2 ratio (Figure 7).

#### 2.1.3. Dynamic Interfacial Tension (IFT)

**Figure 8:**
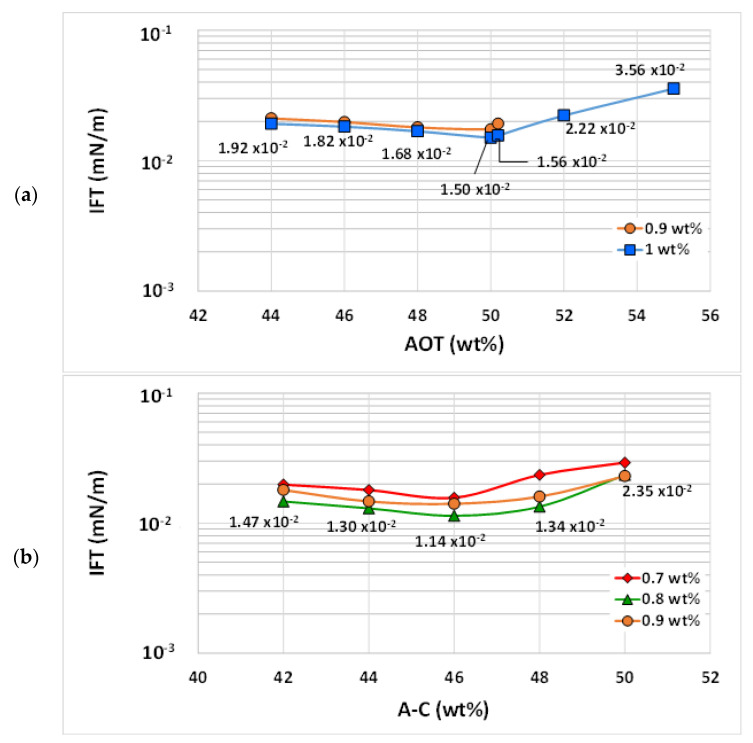
Interfacial tension measurements as a function of blend ratio and concentration at 25 °C for (**a**) AOT/LF2 and (**b**) A-C/LF2 blends.

As part of the optimal blend ratio definition, several ratios considered of interest according to the phase behavior evaluation (Section 2.1.1) and the stability tests (Section 2.1.2) were selected to measure dynamic IFT. Other close concentrations were also considered. Figure 8 shows the equilibrated interfacial tensions measured at 25 °C, as a function of blend ratio and concentration, for the AOT/LF2 (Figure 8a) and A-C/LF2 blends (Figure 8b). 

The lowest IFT value achieved with the AOT/LF2 blend was 1.50 × 10^−2^ mN/m at the 50/50 blend ratio and at 1 wt% concentration in SSW. A slight increase in the IFT values was observed when the blend concentration was reduced to 0.9 wt%. In the case of the A-C/LF2 blend, the lowest IFT value was 1.14 × 10^−2^ mN/m, observed at a 46/54 ratio (the composition in the ternary blend was AOT_20.7wt%_/[C_12_mim]Br_25.3wt%_/LF2_54wt%_) and at 0.8 wt% concentration in SSW. A higher concentration (0.9 wt%) and a lower one (0.7 wt%) were also evaluated, resulting in slightly higher IFT values.

#### 2.1.4. Dynamic Adsorption

**Figure 9:**
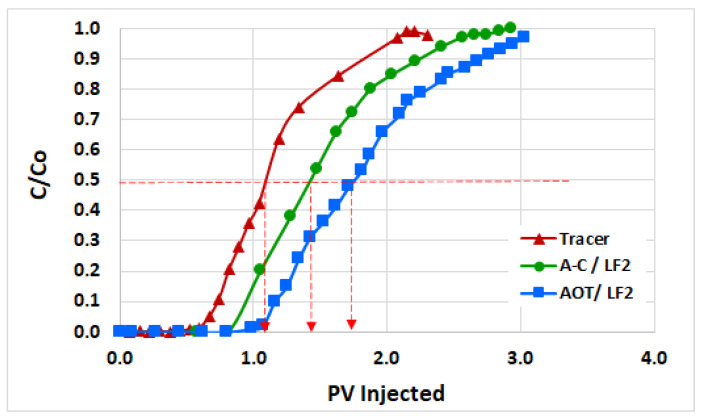
Estimation of dynamic adsorptions for optimal blends AOT/LF2 and A-C/LF2 in carbonate core at room conditions.

The results of the dynamic adsorption tests carried out in carbonate rocks are shown in Figure 9. This figure shows the relative concentrations (*C*/*C*_0_) of the tracer and the optimal blends in the effluent as a function of the injected pore volume (*PV*) at room conditions. The tracer front achieved 50% of the initial concentration (*C*/*C*_0_ = 0.5) at 1.09 *PV* injected, while for the optimal blends AOT/LF2 and A-C/LF2 this concentration ratio was achieved at 1.75 and 1.43 *PV*, respectively. Using Eq. 2, the dynamic adsorptions were estimated as 0.42 mg/g of rock for AOT/LF2 and 0.21 mg/g of rock for the A-C/LF2 blend. 

The lower adsorption of the A-C/LF2 blend compared with AOT/LF2 could be due to the high electrostatic interaction between the oppositely charged head groups of AOT and [C_12_mim]Br, increasing the synergy between them and tending to form ion pairs [32,33] which reduce the free negative charges of AOT. The AOT/LF2 blend is less synergistic than A-C/LF2. An analysis of the AOT/LF2 and AOT/[C_12_mim]Br electrostatic interactions is discussed in our previous study [15].

#### 2.1.5. Core Flooding Tests

In the AOT/LF2 core flooding test, during the water flooding stage, 1.97 *PV* of SSW was injected (until water production reached around 99% of the total production) through the carbonate core at 0.05 mL/min. The oil recovery was 47.7% of OOIP. For the chemical flooding, a slug of around 0.5 *PV* of the optimal formulation (1 wt% of 50/50 AOT/LF2 prepared in SSW) was injected followed by 1.26 *PV* of polymer flooding, both at 0.05 mL/min. The achieved AOR was 7.3% OOIP.

In the case of the A-C/LF2 core flooding test, the core was water flooded at 0.05 mL/min with 1.96 *PV* of SSW (no oil production was observed at that point), and an oil recovery of 40.8% of OOIP was obtained. Then, the core was flooded with 0.51 *PV* of the optimized blend (0.8 wt% of 46/54 A-C/LF2 prepared in SSW) at 0.05 mL/min, followed by 1.38 *PV* of polymer flooding at the same injection rate. The AOR in this case was 11.5% OOIP. The apparent viscosity of the FloPAM 920 SH polymer solution for both core flooding tests at 2000 ppm of concentration and at 25°C was 5.7 mPa S, measured at a shear rate of 10 s^−1^.

**Figure 10:**
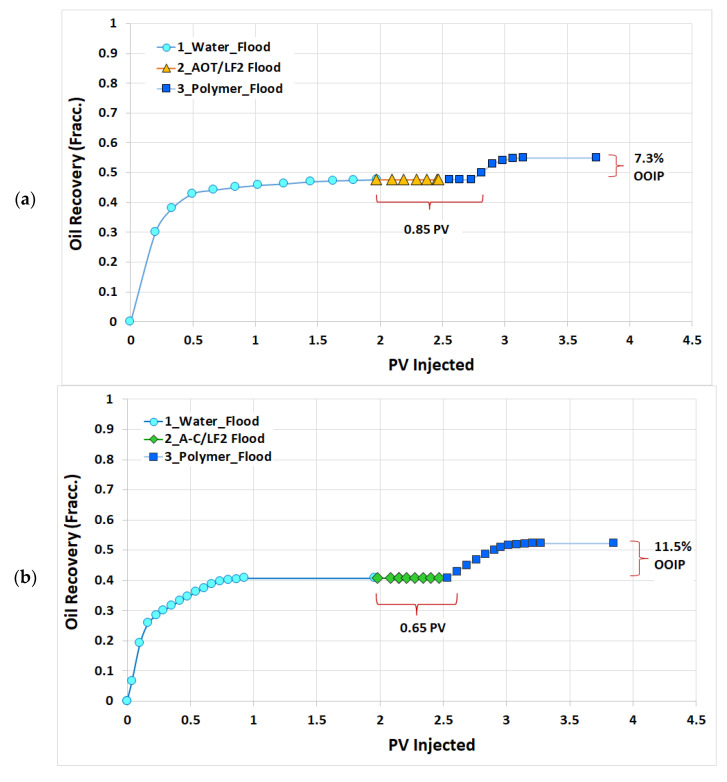
Oil recovery during water, blend and polymer injections in core flooding tests at room temperature: (**a**) AOT/LF2 and (**b**) A-C/LF2.

Table 2 presents a summary of the two core flooding tests, and Figure 10 shows the evolution of the oil recovery during water, surfactant, and polymer flooding. It can be seen that in the case of the AOT/LF2, oil production started after the injection of 0.85 *PV* of chemicals, whereas only 0.65 *PV* was required with A-C/LF2. This might be due to the higher adsorption found with the AOT/LF2 blend (Section 2.1.4).

## 3. Materials and Methods

### 3.1. Experimental

#### 3.1.1. Materials

**Figure 11:**
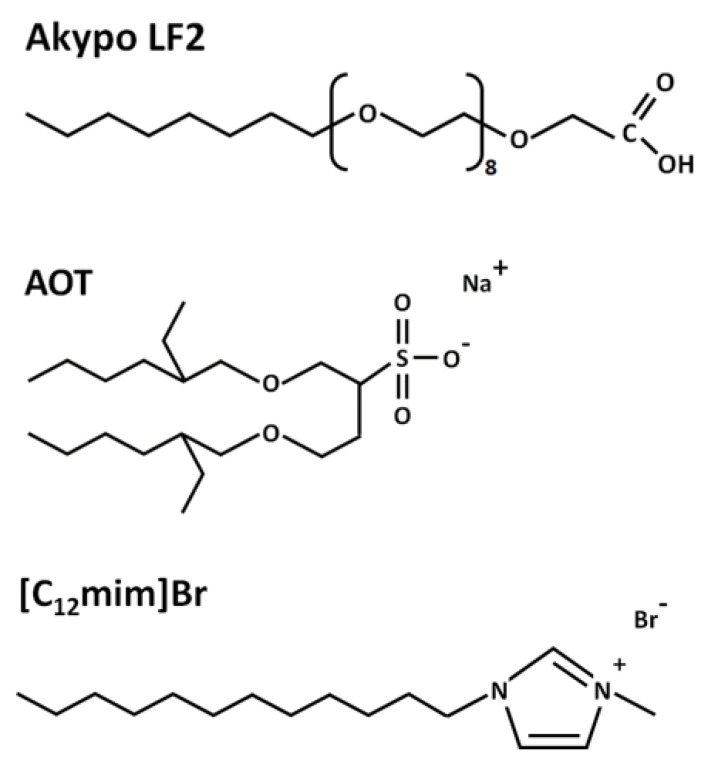
Chemical structure of surfactants and SAIL.

The AOT surfactant, dioctyl sulfosuccinate sodium salt, and potassium iodide (KI) were purchased from Sigma-Aldrich with purities of ≥ 97 wt% and > 99 wt%, respectively. The surfactant polyoxyethylene(8) octyl ether carboxylic acid (C_8_EO_8_OCH_2_COOH), commercially named Akypo LF2, was kindly provided by KAO Chemicals with a purity ≥ 98 wt%. The SAIL 1-dodecyl-3-methylimidazolium bromide ([C_12_mim]Br) was purchased with a purity > 98 wt% from Iolitec. Figure 11 presents the chemical structures of the surfactants and the SAIL. 

Polyacrylamide FloPAM FA 920 SH polymer (6.5–8.5 × 10^6^ Da) was kindly provided by SNF Floerger. Synthetic sea water (SSW) was prepared with salts. Its composition is detailed in Table 3. 

Dead crude oil was kindly supplied by CEPSA. Table 4 shows its main characteristics. Outcrop carbonate rock cores (Indiana Limestone), supplied by Kocurek Industries, were used for the dynamic adsorption and core flooding experiments. The mineral details can be found in a previous work [27].

#### 3.1.2. Methods

Stock solutions of the surfactants AOT, LF2 and the ionic liquid ([C_12_mim]Br were prepared individually in distilled water at 8 wt% concentration, to prevent possible interactions of each component with divalent salts prior to blending them. The required amounts of surfactant solutions were mixed to obtain aqueous formulations with a blend concentration of 4 wt%. Aiming to carry out phase behavior studies, a brine solution twice the concentration shown in Table 3 was added to obtain an aqueous solution of 4 wt% surfactant blend in SSW. Lower blend concentrations for the adsorption and core flooding tests were prepared in a similar way. A Mettler Toledo XPE205 analytical balance was used for the preparation of the solutions by weight. 

##### Blend Scans

To evaluate the phase behavior of the microemulsions and identify the blend ratio corresponding to the optimal formulations (lowest interfacial tension), blend scans were carried out using the encased-glass-pipette methodology [33,34]. The binary and ternary blends were prepared at 4 wt% concentration in SSW (a concentration higher than usual in practice but one that allowed the easy visualization of the phases). The water-oil ratio in the pipettes was about 1:1, with ~1 mL of aqueous blend solution and ~1 mL of crude oil as the oil phase. For AOT/LF2, the blend scan was performed varying the ratio from 0/100 to 100/0 wt%. In the case of the AOT/[C_12_mim]Br/LF2 blend, the AOT/[C_12_mim]Br was considered as a pseudo component (hereinafter termed A-C), so the ratios were changed from 0/100 A-C/LF2 to 100/0 A-C/LF2. Preliminary studies were carried out to fix the composition of A-C to 45 wt% AOT (55 wt% [C_12_mim]Br). This initial work can be found in Figure S1 in Supporting Information. Phase behavior was evaluated at 25 °C, 50 °C and 75 °C using an OVAN dry-block heater (model BD200-RE). 

The oil and aqueous phase volumes were measured in the pipettes to calculate the solubilization parameters for water (*V_w_/V_s_*) and oil (*V_o_/V_s_*), defined as the volume of water (*V_w_*) and the volume of oil (*V_o_*) solubilized in the microemulsion phase per volume of surfactant (*V_s_*), assuming that all the surfactant is present in the microemulsion phase [20,33]. An optimal formulation is obtained when the system shows a middle microemulsion in equilibrium with excess oil (upper) and excess water (lower) phases and the solubilization parameters are equal and large. Interfacial tensions between the aqueous and oleic phases at optimal solubilization parameters (V_i_/V_s_*, when *V_o_/V_s_* = *V_w_/V_s_*) were estimated using the Huh’s correlation [38]:

(1) $$IFT_{Huh}=\frac{C}{{\left({\frac{V_{i}}{V_{S}}}^{*}\right)}^{2}}$$

where *C* = 0.3 mN/m. 

##### Stability Tests

To determine the possible precipitation or separation of the blend’s components, the stability of the blends in the absence of oil was evaluated by preparing 1 wt% blend (a concentration commonly used in the application), with different ratios, in SSW at room temperature. The evaluation was carried out considering the translucence of the solutions [11,27,30]. 

##### Dynamic Interfacial Tension

This evaluation was performed, taking into account the range of interest found in the pipette tests, to precisely determine the optimal blend ratio for both blends. Measurements of the interfacial tension between the aqueous phase and the crude oil were carried out using a Krüss spinning drop tensiometer (model SITE100) at 25 °C, as in our previous reports [27,30]. A drop of 4 μL of the crude oil was left in the middle of the capillary tube filled with the aqueous formulations, and the rotation velocity was set to obtain an oil drop length at least four times larger than its diameter [39,40]. Densities of the aqueous blends were measured with the help of an Anton Paar density meter (model DMA 5000 M). 

##### Dynamic Adsorption

Determination of the adsorption of the blends on carbonate rocks was conducted in a Hassler core holder equipment (model H00-021-0) for each optimal blend through single-phase dynamic adsorption tests at room temperature. The cores were vacuumed for 24 h and then saturated with SSW for 24 h at 0.05 mL/min. Absolute permeabilities (*Ka*) were estimated by Darcy’s law at different injection rates, while pore volumes (*PV*) and porosities (*Ø*) were calculated using wet and dry core weights, their bulk volume, and the SSW density. Potassium iodide (KI) was injected at 0.1 mL/min as the tracer. The effluent was sampled until it achieved the initial KI concentration. The cores were cleaned by injecting SSW until no KI was produced. Then, the optimized blend was injected at 0.1 mL/min. Samples of effluent were taken until they achieved a blend concentration equal to the initial value. The difference between the tracer and the blend fronts (defined as the 50% of their initial concentration in effluents) was used to determine the blend adsorption [20,27,41]:

(2) $$\tau =\frac{\left(PV_{blend,50\%}-PV_{tracer,50\%}\right)\times PV\times {\left[C_{0}\right]}_{blend}}{mass_{rock}}$$

where *τ* is the blend adsorption in mg/g, *PV_blend,_*_50%_ and *PV_tracer,_*_50%_ are the pore volumes in which effluent has reached 50% of initial concentration of blend and tracer, respectively, *mass_rock_* is the dry core weight in grams, *PV* is the pore volume in mL and *[C_0_]_blend_* is the initial blend concentration in mg/mL. To estimate the concentrations of KI and the blend in effluents, an HP UV/Vis-spectrophotometer (model Presario SR1000) was used.

##### Core Flooding Tests

Two core flooding experiments using fresh carbonate cores were performed at room temperature following a similar protocol to that described above for the adsorption test. The cores were initially vacuumed and saturated with SSW at 0.05 mL/min (~1 ft/d) for 24 h. After the estimation of the absolute permeability, pore volume, and porosity, dead crude oil was injected at 0.05 mL/min for 24 h, and the expelled water was used to calculate the original oil in place (OOIP) and the initial water saturation (*S_wi_*). The cores were left to equilibrate for 8 days and then water flooded with SSW at 0.05 mL/min. Volumes of injected water, produced water and produced oil were recorded. Oil recovery was measured during the tests, and the residual oil saturation for water flooding (*S_orw_*) was estimated by material balance. Surfactant flooding was performed by injecting optimized blend formulations at 0.05 mL/min, and finally, polymer flooding was carried out by injecting the polymer PAM 920 SH (2000 ppm) at 0.05 mL/min until no oil was detected in the effluents. Residual oil saturation after chemical flooding (*S_or2_*) and additional oil recovery (AOR) were estimated. The apparent viscosities of the polymer at 25°C as a function of shear rates were measured using an Anton Paar rheometer (model MCR 102).

## 4. Conclusions

In this work, the evaluation of the blend AOT/LF2 for enhanced oil recovery in carbonate reservoirs was carried out, and the improvement of its efficiency by adding the SAIL [C_12_mim]Br to the formulation was also assessed. From this study, some conclusions may be established.

The phase behavior of AOT/LF2 and A-C/LF2 (A-C being a mixture containing 45 wt% AOT and 55 wt% [C_12_mim]Br) in SSW is very similar, leading in both cases to Winsor type III systems of interest for EOR applications. 

A decrease in solubilization parameters with temperature suggests, in both cases, the application of the formulations in reservoirs without excessively high temperatures.

According to phase behavior and IFT studies, the best formulation with the binary blend consists of 1 wt% of AOT_50wt%_/LF2_50wt%_ blend in SSW. In the case of the ternary blend, the optimal formulation contains 0.8 wt% of AOT_20.7wt%_/[C_12_mim]Br_25.3wt%_/ LF2_54wt%_.

Both blends are stable without oil and in the presence of divalent ions, thus ensuring their injectability in surfactant flooding.

When the SAIL is added to the formulation, the electrostatic interactions between the oppositely charged surfactants (AOT and [C_12_mim]Br) led to a greater reduction in the water–oil IFT and lower adsorption of the blend on carbonate rocks.

Tertiary oil recovery with the ternary blend increases the AOR achieved with the binary blend by 57.5% (in both cases surfactant being followed by polymer flooding), pointing to the former as most promising for the application.

## Figures and Tables

**Table 1:** Optimal blend ratios, solubilization parameters and IFT_Huh_ at 25 °C and 50 °C, for AOT/LF2 and A-C/LF2 blends.

	AOT/LF2		A-C/LF2	
Temperature	25 °C	50 °C	25 °C	50 °C
Optimal blend ratio	50.2/49.8	55/45	49.9/50.1	40.7/59.3
V_i_/V_s_*	3.0	2.1	4.7	2.5
IFT_Huh_ (mN/m)	3.36 × 10^−2^	6.64 × 10^−2^	1.32 × 10^−2^	4.87 × 10^−2^

**Table 2:** Results of the core flooding experiments.

Formulation		
Blend Composition	AOT (50 wt%) LF2 (50 wt%)	AOT (20.7 wt%) [C_12_mim]Br (25.3 wt%) LF2 (50 wt%)
Concentration in SSW (wt%)	1	0.8
**Initial conditions**		
Porosity, *ø*	0.136	0.18
Permeability, *K_a_* (mD)	16.81	19.16
Pore volume, *PV* (mL)	12.03	15.6
Oil visc. (mPa S) at 25 °C	12	12
OOIP (mL)	6.80	8.20
Initial water saturation, *S_wi_*	0.43	0.47
**Water Flooding**		
Injection rate, *Q_i_* (mL/min)	0.05	0.05
Injected SSW Volume (*PV*)	1.97	1.96
Oil recovery (%OOIP)	47.7	40.8
Residual oil saturation, *S_orw_*	0.36	0.37
**Chemical Flooding**		
Injection rate (*Q_i_*, mL/min)	0.05	0.05
Injected blend volume (*PV*)	0.495	0.511
Injected polymer volume (*PV*)	1.26	1.38
AOR (%OOIP)	7.3	11.5
Residual oil saturation, *Sor*_2_	0.33	0.32

**Table 3:** Synthetic sea water (SSW) composition.

Salt	Purity (wt%)/ Commercial	SSW (g/L)
Na_2_SO_4_	>99%/Sigma-Aldrich	4.84
CaCl_2_·2H_2_O	>99%/Sigma-Aldrich	1.89
MgCl_2_·6H_2_O	>99%/Sigma-Aldrich	15.06
NaCl	>99%/Panreac	27.94
TDS (g/L)		49.73
Density at 25 °C (g/mL)		1.028

**Table 4:** Main properties of crude oil.

Property	Crude Oil A
Density at 25 °C (g/mL)	0.853
°API	34.1
Viscosity at 25 °C (mPa·s)	15.3
Saturates (wt%)	61
Aromatics (wt%)	33
Resins (wt%)	4.6
Asphaltenes (wt%)	1.4

## Data Availability

Not applicable.

## Supplementary Materials

The following supporting information can be downloaded at: https://www.mdpi.com/article/10.3390/ijms24010726/s1.
