# Supplementary material for: Improvement of a Surfactant Blend for Enhanced Oil Recovery in Carbonate Reservoirs by Means of an Ionic Liquid
Source: Int J Mol Sci. 2022 Dec 31;24(1):726. doi: 10.3390/ijms24010726 (PMC9821681; doi:10.3390/ijms24010726)
Supplement: Supplementary file 1 [file ijms-24-00726-s001.zip › ijms-2100322-supplementary.pdf]

Supporting Information

Improvement of a Surfactant Blend for Enhanced Oil Recovery in Carbonate Reservoirs by Means of an Ionic Liquid

Nestor Tafur [1], Alberto P. Muñuzuri [2] and Ana Soto [1,*]

[1] CRETUS, Department of Chemical Engineering, Universidade de Santiago de Compostela, 15782 Santiago de Compostela, Spain
[2] Group of Nonlinear Physics, Galician Center for Mathematical Research and Technology (CITMAga), 15782 Santiago de Compostela, Spain
* Correspondence: ana.soto@usc.es

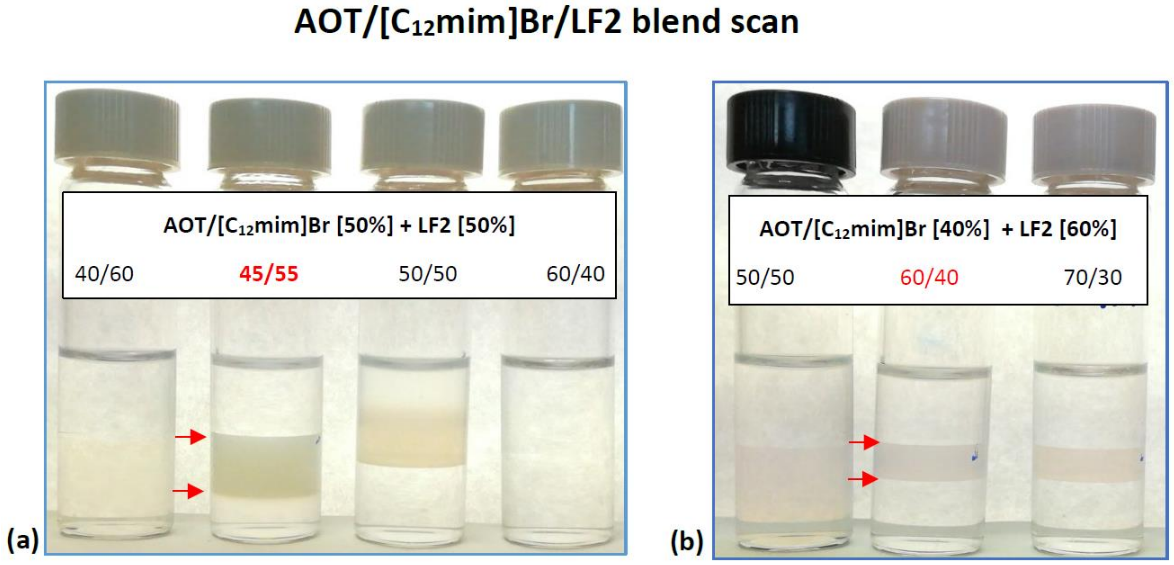

Figure S1. AOT/[C12mim]Br + LF2 blend scan as a function of AOT/[C12mim]Br ratio. (a) AOT/[C12mim]Br [50%] + LF2 [50%]. (b) AOT/[C12mim]Br [40%] + LF2 [60%]. Test temperature 25°C. N-octane as oil phase.
